# On biochemical constructors and synthetic cells

**DOI:** 10.1098/rsfs.2023.0014

**Published:** 2023-08-11

**Authors:** Sebastian J. Maerkl

**Affiliations:** Institute of Bioengineering, School of Engineering, Ecole Polytechnique Federale de Lausanne (EPFL), Lausanne, Vaud, Switzerland

**Keywords:** synthetic cell, biochemical constructor, cell-free synthetic biology

## Abstract

Is it possible to build life? More specifically, is it possible to create a living synthetic cell from inanimate building blocks? This question precipitated into one of the most significant grand challenges in biochemistry and synthetic biology, with several large research consortia forming around this endeavour in Europe (European Synthetic Cell Initiative), the USA (Build-a-Cell Initiative) and Japan (Japanese Society for Cell Synthesis Research). The mature field of biochemistry, the advent of synthetic biology in the early 2000s, and the burgeoning field of cell-free synthetic biology made it feasible to tackle this grand challenge.

Building a synthetic cell is an enormous task, despite the considerable knowledge accumulated by biochemists and biologists over decades of work, who laid the foundational knowledge required to understand the function of individual biological components and their interplay in a cellular environment. Incidentally, many of the seminal discoveries and characterizations of biochemical components and systems were originally conducted in cell-free environments *in vitro* [1]. Synthetic biology injected engineering into biology and drastically improved our ability to design and build increasingly complex biological systems. Cell-free synthetic biology, in turn, has made it possible to engineer biochemical systems *in vitro*, eliminating the confounding complexity inherent with cellular engineering. Together with developments in the art of performing complex manipulations of minute volumes of liquid using sophisticated microfluidic devices and the ability to generate cell-like materials such as lipid bilayers kindled the exciting possibility to assemble a living synthetic cell from inanimate biochemical building blocks [2].

But what is life exactly? A deceivingly simply question without a definitive answer, although a certain consensus exists on the core functions a living system needs to fulfil: (i) self-replication: the ability to generate copies of itself, (ii) evolvability: the ability to undergo Darwinian evolution, and (iii) adaptability: the ability to sense and adjust to changing environments [3,4]. It could be argued that only self-replication and evolvability are required as all other functions, including adaptability, could in theory evolve over time in a self-replicating evolvable system. One naturally considers cells and multicellular organisms as the reference for what constitutes a living system. Cells have the additional hallmark of being compartmentalized, although compartmentalization might not be a strict necessity for a living biological system considering that the origin of life may have arisen in a non-compartmentalized environment (or at least not in a compartment created by a lipid bilayer) [5]. Nonetheless, the ultimate goal of building a synthetic cell will most probably culminate in the creation of a compartmentalized biochemical system that is capable of self-replication, evolution and adaptation.

Is it possible to build a synthetic cell? Building a synthetic cell requires the functional implementation of several complex biochemical systems and consequent integration of these. Self-regeneration is a central capability to be achieved [6]. All essential macromolecular components including non-ribosomal proteins [6–8], ribosomal proteins and RNA [9] and transfer RNAs [10] will have to be functionally synthesized. In addition, DNA replication needs to be achieved [11,12], and a rudimentary metabolism may need to be implemented [13,14], including the ability to synthesize lipids to create compartmentalization [15]. It is likely that the mechanisms for cell fission have to be re-created [16], although it might be possible to circumvent this requirement through physical shearing of larger vesicles into smaller ones [5].

Exactly what level of complexity has to be achieved for a functional synthetic cell to be established is unknown. We have some indication of the upper bound in complexity needed to create a synthetic cell based on organisms such as *Mycoplasma genitalium* (475 genes) [17] and *Pelagibacter ubique* (1354 genes) [18], which have the smallest genomes known for an obligate pathogen and a free-living organism, respectively. Similarly, the last universal common ancestor (LUCA) is thought to contain a similar number of approximately 500–600 genes [19]. One of the smallest known obligate symbionts is *Nasuia deltocephalinicola* which requires only 137 genes for life [20]. 100–200 genes are becoming tractable with today's state-of-the-art techniques and methods, and may be sufficient for life [21].

Nonetheless, complexity remains enormous, even if only as few as approximately 130 genes are required to build a functional synthetic cell. In order to further reduce complexity, it is worthwhile pondering what the absolute minimal requirement for a self-replicating biochemical system might be. John von Neumann tackled a similar question in a Gedanken experiment in which he derived the concept of a universal constructor in the 1940s [22]. Neumann was interested in understanding what the minimal requirements were to create a machine capable of self-replication. Neumann's universal constructor consists of three parts: a constructor, a copy machine and an instruction tape. When placing a fully functional universal constructor in a space surrounded by freely moving parts, the universal constructor could assemble these parts according to the instruction tape into a replica of itself. The instruction tape would be copied by the copy machine and the copy inserted into the newly constructed machine creating an exact functional copy of the original, which in turn would be capable of creating a functional replicate of itself. If errors are introduced during the process of copying the instruction tape, the system could in principle evolve. Neumann also pointed out that the complexity of the building blocks available for assembly is critically important, as the problem would either be trivialized by the availability of parts that are too complex, or become intractable if the parts are too basic. The extremes being that the available parts are the constructor and copy machine themselves, requiring no further assembly other than combining these two machines. On the other end of the spectrum, one could imagine raw materials as input, which would require a series of complex manipulations to assemble into basic components, which themselves require further downstream assembly.

To date it has not been possible to create a physical implementation of Neumann's universal constructor, but the concept has been implemented *in silico* [23]. Interestingly, and most impressively, Neumann derived the central processes living organisms use to attain life well before the biochemical details of biological self-replication and information transfer were known, and Neumann's universal constructor thus strongly influenced biochemists at the time. Once biology caught up, it became clear that Neumann's vision of a universal constructor was strictly analogous to living systems. The constructor being analogous to proteins, ribosomes, and other large macromolecules required for self-regeneration through the central dogma, the copy machine being analogous to DNA polymerase, the instruction tape being analogous to DNA, and the parts being analogous to small molecule building blocks such as amino acids and nucleotides required for assembly of the larger macromolecules such as proteins, DNA and RNAs. Biology thus has most impressively achieved the implementation of Neumann's universal constructor.

One can thus consider whether a universal biochemical constructor could be implemented as a viable path towards creating a living biochemical system. For brevity the term ‘biochemical constructor’ will be used throughout this text, while always implicitly referring to a universal biochemical constructor. It should be feasible to implement a biochemical constructor on a microfluidic chemostat [24] using the recombinant cell-free PURE system [25]. The PURE system is capable of executing the central dogma (transcription and translation) and is generated by reconstituting purified biomolecules including approximately 36 proteins, ribosomes and tRNAs.

The issue of choosing the correct level of building block complexity is simpler for biological systems, as biochemical building blocks are well defined and minimal. Unlike the example of the universal constructor where use of low-complexity building blocks very quickly renders the realization of a universal constructor intractable, use of simple building blocks in a biochemical constructor is quite straightforward. Furthermore, these building blocks and the biochemical constructor itself do naturally exist in a sea of freely moving parts, which is another major fundamental problem for creating an electro-mechanical von Neumann type universal constructor. A range of possibilities of course exists, as biological systems can generate biomass from very simple building blocks, but many organisms exist that strongly depend on more complex building blocks including amino acids and nucleotides. Extreme examples of the latter are the obligate symbionts such as *N. deltocephalinicola* briefly mentioned above, which retain only a very limited metabolism.

The PURE system in principle might be able to self-replicate all of its components including its 36 non-ribosomal proteins, ribosomal proteins, ribosomal RNAs and tRNAs. When combined with DNA replication it should then, at least in theory, be possible to self-replicate the PURE system and a synthetic genome encoding it, creating a biochemical constructor that can be programmed to achieve adaptability, and meeting two of the three central hallmarks of life. It is unclear whether it will be possible to implement evolvability, which likely will require sub-compartmentalization. Such a system would be very similar to an obligate symbiont which heavily relies on its host to sustain it with small molecule building blocks. In 2020, we were able to demonstrate a proof-of-concept by robustly and sustainably self-regenerating up to seven essential proteins over extended periods of time in the PURE system running on a microfluidic chemostat device [6,24]. Achieving a fully self-replicating biochemical constructor remains an enormous challenge. All approximately 36 non-ribosomal proteins will have to be self-regenerated [6,7,26], tRNAs will have to be regenerated [10] and DNA replicated [12,27]. But these have been shown to be feasible in isolated experiments. Achieving sustained and full self-regeneration of any of these sub-systems remains challenging, not to mention further integration of these into a fully self-regenerating system. Another significant challenge will be ribosome self-regeneration. Although individual ribosomal components can be synthesized *in vitro*, functional assembly of a ribosome remains a major challenge in the field [9].

These recent advances indicate that creating a biochemical constructor may indeed be feasible, but also obviated several technical challenges. One of the major current problems is the limited synthesis rate of the PURE system. We estimated that the synthesis rate of the PURE system running at steady state on a microfluidic chemostat device is 25 times below the rate required to synthesize all non-ribosomal proteins in the PURE system, and this estimate excluded ribosomal proteins, rRNAs and tRNAs. This problem can potentially be at least partially solved by adjusting the dilution rates used when running the PURE system on a chemostat. Current dilution rates are rather high, approaching the doubling time of fast-growing *E. coli*. A lower dilution rate would mean that fewer macromolecular components have to be synthesized per unit time. We recently explored whether it is possible to lower dilution rates while maintaining the synthesis rate of the PURE system. Simply lowering dilution rates led to a decrease in overall synthesis rate. This occurred because the PURE system when run at steady state could only sustain synthesis for a relatively short period of time likely due to limited resource availability. This problem in turn could be solved through the inclusion of semi-permeable membranes that permit diffusion of small molecule building blocks while preventing dilution of larger macromolecular components [28]. This improved chemostat device now permits running the PURE system at lower dilution rates without incurring losses in synthesis rate. A second approach to solving the problem of limited synthesis rate is to optimize the PURE system itself. Although considerable efforts have been made to optimize PURE system composition, these efforts have thus far focused on improving the total synthesis capacity of the system [29–31]. Rather than optimizing total protein synthesis capacity, we posit that for the purpose of building a biochemical constructor the PURE system should be optimized differently. We considered whether the PURE system could be optimized by maintaining or even enhancing the ratio of protein synthesis rate to total protein content in the PURE system. This appeared to be a promising approach based on some relatively small optimization steps we recently implemented [6], and current efforts indicate that further improvements can be achieved in this direction.

One main concern in the field was the question as to whether the PURE system could functionally synthesize its components. Recent work on the synthesis of aminoacyl tRNA synthetases (aaRSs) and tRNAs produced *in vitro* by the PURE system seems to largely alleviate this concern, as it was demonstrated that all aaRSs except PheRS could be functionally synthesized by the PURE system [32]. It was likewise recently demonstrated that tRNAs could be *in vitro* transcribed and used in the PURE system, although this approach used a separate *in vitro* transcription step performed on modified DNA templates, the addition of RNase P, and purification prior to addition of the *in vitro* transcribed tRNAs to the PURE system [10]. Nonetheless, despite being far from ideal, these experiments indicate that synthesis of all non-ribosomal PURE components may be within reach. Ribosome biogenesis on the other hand represents a challenge, and significant progress in synthesizing and assembling functional ribosomes *in vitro* has yet to be achieved. Similar to the question as to whether PURE could synthesize its own components, it was also not clear whether DNA replication could be implemented in the PURE system. Recent work in this direction has been very promising, demonstrating that DNA replication can be achieved on both linear as well as circular DNA templates in the PURE system [8,12,33]. Although DNA amplification is extremely well established, it has proven difficult to achieve DNA replication in the PURE system, which required considerable reaction condition optimization, indicating that there is a surprising incompatibility between conditions optimal for transcription–translation, and DNA synthesis. Cells may have solved this problem by temporally compartmentalizing these various functions, as reported in the eukaryote *S. cerevisae* [34], although it is unclear when this temporal compartmentalization arose. Initial attempts will therefore likely continue to focus on implementing concurrent DNA replication and transcription–translation *in vitro*, although it may be possible to spatially, if not temporally, segregate these processes if necessary.

Attempts to self-regenerate PURE system components and DNA replication thus far have not implemented regulation. When self-regenerating aaRSs we found that expression levels of this class of enzymes were relatively robust in that a minimal expression level needed to be maintained for achieving optimal protein synthesis rates [6]. Nonetheless, under-expression led to significant decreases in proteins synthesis rate, likely due to rate limitations arising from insufficient tRNA aminoacylation. Likewise, overexpression led to a decrease in synthesis rates, likely due to now well-described loading effects on the system [35]. Self-regeneration of T7 RNA polymerase on the other hand was extremely sensitive to expression levels, exhibiting a narrow range of optimal concentrations [6]. We suggested that in addition to creating loading effects when overexpressed, T7 RNAP overexpression also creates considerable imbalances in resource allocation through the synthesis of excess mRNA, which leads to a reduction in translation capacity and thus an overall lower protein synthesis rate. It is therefore likely that implementation of regulatory mechanisms can improve both overall protein synthesis capacity as well as entail robustness. A plethora of regulatory mechanisms have been shown to be functional in cell-free systems including transcriptional regulation, post-transcriptional regulation and targeted protein degradation [24]. Complex transcriptional regulatory networks have been engineered using both native [36] and synthetic transcription factors [37], as well as dCas9-based regulation [38]. Riboswitches and tRNA amber stop codons [24] have been used for post-transcriptional regulation, and target protein degradation was achieved using an *in vitro* synthesized ClpXP protease [24]. Using these tools, it will be interesting to de novo engineer control systems [39] to precisely control expression levels of various components of the PURE system and to ultimately encode all genes on a single, de novo designed PURE genome. The latter will require a precise understanding of how to encode and implement large systems *in vitro*.

The PURE system itself is very close to an ideal starting point to the development of a biochemical constructor as it is quite well defined and consists of a tractable number of components [25] as compared to lysate-based alternatives, which are highly complex. Although the PURE system is close to ideal, there are several caveats associated with its use. Generating the PURE system requires individual purification of 36 protein components as well as the ribosomes, which is a rather cumbersome process, but it entails full flexibility in terms of adjusting PURE system composition including adjusting concentrations of individual components and drop-out recipes in which one or more of the PURE components is omitted. The latter is critical for performing self-regeneration experiments. To simplify the process, we developed a method that allows the concurrent purification of all 36 non-ribosomal protein components in a single purification step. This OnePot PURE method renders the system more readily accessible for research and industrial applications, while permitting the creation of various PURE formulations, as well as creating PURE systems lacking one or more components, albeit with decreased precision over the exact concentration of each component [40]. Maybe one of the major caveats of the PURE system when used in self-regeneration experiments is the fact that it is not entirely pure. Although most PURE components should in theory be essential [41], it has become clear that impurities in the PURE system render certain essential dropouts non-essential, making it potentially difficult to ascertain whether functional self-regeneration has indeed been achieved. Care must therefore be taken to ascertain that individual components being self-regenerated are indeed essential [6]. This problem is mainly affecting aaRSs, which should in theory be all essential, but trace amounts of contaminating aaRSs appear to recover functional protein synthesis. This effect of contamination will also strongly depend on the purification quality achieved for the individual components, and impurities may not only be contained in the various his-tag purifications of the non-ribosomal protein components, but also likely originate from the ribosome purification. Impurities are an annoying technical problem, especially for experiments demonstrating self-regeneration. In the long term, it is likely that impurities will decrease if not be entirely eliminated as more and more components can be successfully self-regenerated from non-contaminated DNA templates.

Are there any fundamental limits that could prevent the realization of a biochemical constructor or synthetic cell? Life is ubiquitous, and low-complexity species such as obligate symbionts have been identified, and it is likely that even simpler life forms remain to be discovered. It therefore appears to be not so much a question of whether synthetic life can be created but rather a question of how synthetic life can be achieved. One of the primary concerns in this endeavour is the question of what level of complexity has to be achieved for synthetic life to be created. A second fundamental worry is whether the components being used for creating a synthetic cell are compatible with this endeavour as they are generally derived from highly complex species such as *E. coli*, which consists of over 4400 genes. All of these components have evolved to function in a complex environment, and may require cofactors and other components for proper function. Furthermore, all *in vitro* environments used to create a biochemical constructor or synthetic cell are considerably more dilute compared to the physiological *in vivo* environments. It is thus a valid concern as to whether it will be possible to create a functional system using such components and deploy them in a substantially different physical environment. Despite these concerns, it is maybe easy to overlook that biochemistry has been very successful in implementing complex biochemical processes in low-complexity environments that have very little similarity with the native environments these systems normally function in. Central processes including transcription and translation can readily be implemented *in vitro*. It therefore appears that biochemical components are surprisingly robust and their function is not entirely co-dependent on the inherent complexities of the environments that they originate from and nominally function in.

Creating a biochemical constructor or synthetic cell from the bottom up is also a quite different challenge from the somewhat related attempt of creating a minimal cell using top-down approaches [42]. With top-down approaches, one starts with a living system and systematically minimizes its gene content. The biggest caveat of top-down approaches is that the final goal is rather nebulous. It will be extremely difficult to claim success as it is technically challenging to prove that a minimal cell is indeed minimal. To definitively show that a minimal cell has been achieved would require a vast combinatoric gene deletion analysis, and even if such a system is shown to be minimal, it will be only a particular solution that strongly depends on the original choice of cell being minimized. Nonetheless, impressive progress has been made on this front, particularly through the demonstration that a synthetic genome could be transplanted into the cytoplasm of a different cellular background, which then led to the complete conversion of one species to another, indicating that kick-starting or booting of synthetic genomes is indeed possible [43].

Assuming a biochemical constructor can be achieved, would it also be alive? If a biochemical constructor can be implemented it will achieve one of the main hallmarks of life which is self-replication. A biochemical constructor will be able to take small molecule building blocks and assemble these to self-replicate. In principle a biochemical constructor running in a chemostat device should be able to sustain itself ad infinitum and, in the process, generate functional PURE that can be used to seed additional biochemical constructors. A biochemical constructor can also be programmed to execute more advanced functions that allow it to sense and adapt to changing conditions, such as changes in dilution rate or temperature, rendering it more robust, and by doing so address another hallmark of life. Realizing these functionalities, self-replication and adaptability, would be a major step towards the creation of a living system, but do nonetheless fall short of a system that could be considered alive. One critical function will be evolvability, which will be difficult to achieve in a biochemical constructor despite the fact that DNA replication and error generation provide some but not all of the ingredients for evolution. Initial implementations of a biochemical constructor will likely be polyploid, which in of itself does not eliminate the possibility for evolution but will render the process more difficult. More problematic is the fact that a single biochemical constructor will not evolve improved functionality, but rather devolve into a poorly functioning or non-functioning state over time. This could potentially be solved if it is possible to engineer a system that runs many biochemical constructors in parallel and allows competition between individual constructor entities. Interesting hints at how this could potentially be achieved were demonstrated with a system of interconnected passive chemostats developed for the purpose of studying signal propagation [44]. A clever design of interconnected chemostats may at the very least avoid cessation of function, as fully functioning biochemical constructor entities could take over and re-seed chemostats in which the biochemical constructor has devolved. Another work-around might involve the creation of a virtual feedback loop that monitors all biochemical constructors running on an integrated platform. Use of a virtual feedback loop would entail the ability to detect malfunctioning chemostats, allowing them to be re-seeded. It furthermore would permit selective pressures to be defined so that individual biochemical constructor entities could be selected for improved functions and thus evolve towards higher fitness. These will be interesting and potentially useful additions to the core biochemical constructor functionality, but also require extensive external control over the system. It is thus possible that a biochemical constructor may achieve self-replication, evolvability and adaptability, but not in a fully autonomous manner. The next step will therefore likely require deploying biochemical constructors in smaller compartmentalized systems more closely mimicking cellular systems and to continually improve the system towards a living synthetic cell.

Assuming a biochemical constructor can be created in a chemostat device, the next logical step will be to implement such a biochemical constructor in a more cell-like compartmentalized system. The material used for compartmentalization should allow for dynamic growth and splitting of these compartments. Lipid bilayers seem an obvious choice. Compartmentalization of a biochemical constructor in unilamellar vesicles would be a significant step towards the realization of a synthetic cell that could attain the three main hallmarks of life, but the use of unilamellar vesicles also gives rise to additional challenges and problems. First, it will become necessary to develop and implement a lipid synthesis pathway to allow the generation of additional membranes necessary for continuous expansion and growth [15]. Creation of compartmentalized systems also eliminates one of the main advantages of working with cell-free systems, which is that components can easily be added or removed from these systems, but as discussed above, this can also give rise to problems which led to the creation of a chemostat device that uses an integrated perfusion membrane to allow small molecules to enter the system while preventing macromolecules from diffusing out. Similar mass transport challenges will likely need to be addressed once cell-free systems are being compartmentalized. The use of membrane channels and transporters seems to be the most likely approach towards circumventing this problem [45,46] although engineering vesicle fusion may also be a viable work-around. Finally, compartmentalization in such system might necessitate the implementation of at least a rudimentary vesicle fission mechanism [16].

But why concern ourselves with building a cell at all? Cells are ubiquitous and over decades researchers developed methods and technologies to culture and manipulate them. Nowadays, cells form the core of essentially all of biotechnology and are being engineered for therapeutic applications, and will likely be increasingly used in diagnostic applications in the near future. But there are significant limitations and difficulties associated with the use of natural cells for biotechnology, therapeutic and diagnostic applications. For example, metabolic engineering has made considerable progress [47,48], but engineering complex metabolic networks remains arduous and challenging for several reasons. First, cells have not evolved to perform the functions we would like them to perform, requiring extensive re-wiring of existing metabolic pathways, and product yields often remain limited. Second, implementation of exogenous synthetic systems in a cellular chassis is always fragile as cells find a way to deactivate the exogenous network. It is difficult to foresee all potential impacts the ability to create living synthetic cells will have, but synthetic cells have the potential to supersede the use of natural or engineered cells in product synthesis as well as the development of entirely novel functionalities that could permit synthetic cells to be deployed as sentinels in the environment or the human body. The prospect of integrating synthetic cells with electronic systems is also exciting, and could give rise to systems that combine the best of these two worlds, which is sensing and synthesis for biological systems and information processing for electronics. This could create a new class of bio-electronic devices that can improve how systems sense and interact with their environment. Aside from practical applications, the process of learning to create a synthetic cell will provide fundamental insights into cellular function and will improve our ability to manipulate and engineer naturally occurring cells as well as create sophisticated synthetic systems.

Cell-free systems are already increasingly used in therapeutic and diagnostic applications. Recently, cell-free systems were developed for the rapid detection of viruses [49], as well as for environmental monitoring of toxic compounds such as heavy metals [50]. A classical application of cell-free systems is product synthesis and cell-free systems have been engineered that can create a variety of products ranging from therapeutic antibodies, antimicrobials, to organic molecules [51,52]. Finally, cell-free systems can perform chemistries and reactions that are impossible to achieve in natural cells either because the products generated are toxic to the host cell, or because the host cell biochemistry is limited. For example, it is much easier to incorporate non-natural amino acids into proteins using cell-free systems than with natural cells [53]. Cell-free systems also enable extremely rapid design–build–test cycles facilitating molecular engineering [36] and may provide sustainable solutions for the synthesis and recycling of biomaterials [54].

The creation of a synthetic cell is more than an exercise in developing new and useful technological capabilities. It is a fundamentally curiosity-driven question. In classical and modern culture, there always existed an intrinsic fascination with creating animate or even sentient beings from inanimate objects. Examples of this include the Golem which is described in Jewish folklore as early as the twelfth century, Mary Shelly's nineteenth century story of Frankenstein which continues to captivate our imagination, and the story of Pinocchio is yet another well-known tale in which Geppetto carves a wooden puppet which then magically attains sentience. These fictional ideas are rapidly becoming reality. Extraordinarily sophisticated and life–like robots have been created, capable of moving through complex environments with uncanny dexterity and agility. Artificial intelligence is progressing so rapidly that it is becoming increasingly difficult for us to discern reality from computer-generated graphics, text, and speech. Infusing sophisticated robots with artificial intelligence will likely soon lead us to question when artificial sentience has been attained. What these examples do not address is the question of life itself, unless an artificial being will be able to function also as a universal constructor as stipulated by Neumann, which then would entail it with the ability to self-replicate and evolve. On the other hand, the biochemical constructor and synthetic cells discussed here tackle the central question of life, self-replication and evolution, but at the same time are far removed from anything that could be regarded as sentient. Nonetheless, given the rapid progress in these areas, the twenty-first century may very well see the creation of life forms and sentient entities that did not arise through Darwinian evolution.

## Data Availability

This article has no additional data.
